# Site-Specific Recombination at XerC/D Sites Mediates the Formation and Resolution of Plasmid Co-integrates Carrying a *bla*_OXA-58_- and Tn*aphA6*-Resistance Module in *Acinetobacter baumannii*

**DOI:** 10.3389/fmicb.2018.00066

**Published:** 2018-01-26

**Authors:** María M. Cameranesi, Jorgelina Morán-Barrio, Adriana S. Limansky, Guillermo D. Repizo, Alejandro M. Viale

**Affiliations:** Instituto de Biología Molecular y Celular de Rosario (IBR), Departamento de Microbiología, Facultad de Ciencias Bioquímicas y Farmacéuticas, CONICET, Universidad Nacional de Rosario (UNR), Rosario, Argentina

**Keywords:** *Acinetobacter baumannii*, *bla*_OXA-58_, carbapenem resistance, XerC/D site-specific recombination, antimicrobial resistance plasmids

## Abstract

Members of the genus *Acinetobacter* possess distinct plasmid types which provide effective platforms for the acquisition, evolution, and dissemination of antimicrobial resistance structures. Many plasmid-borne resistance structures are bordered by short DNA sequences providing potential recognition sites for the host XerC and XerD site-specific tyrosine recombinases (XerC/D-like sites). However, whether these sites are active in recombination and how they assist the mobilization of associated resistance structures is still poorly understood. Here we characterized the plasmids carried by *Acinetobacter baumannii* Ab242, a multidrug-resistant clinical strain belonging to the ST104 (Oxford scheme) which produces an OXA-58 carbapenem-hydrolyzing class-D β-lactamase (CHDL). Plasmid sequencing and characterization of replication, stability, and adaptive modules revealed the presence in Ab242 of three novel plasmids lacking self-transferability functions which were designated pAb242_9, pAb242_12, and pAb242_25, respectively. Among them, only pAb242_25 was found to carry an adaptive module encompassing an IS*Aba825*-*bla*_OXA-58_ arrangement accompanied by a Tn*aphA6* transposon, the whole structure conferring simultaneous resistance to carbapenems and aminoglycosides. Ab242 plasmids harbor several XerC/D-like sites, with most sites found in pAb242_25 located in the vicinity or within the adaptive module described above. Electrotransformation of susceptible *A. nosocomialis* cells with Ab242 plasmids followed by imipenem selection indicated that the transforming plasmid form was a co-integrate resulting from the fusion of pAb242_25 and pAb242_12. Further characterization by cloning and sequencing studies indicated that a XerC/D site in pAb242_25 and another in pAb242_12 provided the active sister pair for the inter-molecular site-specific recombination reaction mediating the fusion of these two plasmids. Moreover, the resulting co-integrate was found also to undergo intra-molecular resolution at the new pair of XerC/D sites generated during fusion thus regenerating the original pAb242_25 and pAb242_12 plasmids. These observations provide the first evidence indicating that XerC/D-like sites in *A. baumannii* plasmids can provide active pairs for site-specific recombination mediating inter-molecular fusions and intra-molecular resolutions. The overall results shed light on the evolutionary dynamics of *A. baumannii* plasmids and the underlying mechanisms of dissemination of genetic structures responsible for carbapenem and other antibiotics resistance among the *Acinetobacter* clinical population.

## Introduction

*Acinetobacter baumannii* represents nowadays a significant cause of healthcare-associated infections generally affecting immunocompromised and severely-injured patients with the global spread of a number of epidemic clonal complexes (CC) displaying multidrug-resistance (MDR) phenotypes (Peleg et al., 2008; Roca et al., 2012; Antunes et al., 2014; Wong et al., 2017). MDR strains contain an arsenal of antimicrobial resistance determinants some located in chromosomal resistance islands and others in plasmids, and have shown an outstanding ability to rapidly acquire additional resistance when confronted to new antimicrobials (Peleg et al., 2008; Bertini et al., 2010; Roca et al., 2012; Ramírez et al., 2013; Antunes et al., 2014; Touchon et al., 2014; Nigro et al., 2015; Blackwell and Hall, 2017; Wong et al., 2017). It is in this context that the increasing resistance among MDR *A. baumannii* strains to last-resort therapeutic options such as the carbapenem β-lactams represents a most worrisome problem (Peleg et al., 2008; Roca et al., 2012; Antunes et al., 2014; Wong et al., 2017).

Carbapenem resistance in *A. baumannii* clinical strains results from the interplay of different factors that include as the main cause the acquisition of β-lactamases endowed with carbapenemase activity (Poirel and Nordmann, 2006; Peleg et al., 2008; Poirel et al., 2010; Mussi et al., 2011; Ravasi et al., 2011; Roca et al., 2012; Antunes et al., 2014; Moran-Barrio et al., 2017; Wong et al., 2017). The most frequent acquired carbapenemases in *A. baumannii* are the carbapenem-hydrolyzing class-D β-lactamases (CHDL) OXA-23, OXA-40/24, and OXA-58 and associated variants, whose respective *bla*_OXA_ genes are embedded in distinct plasmid-borne genetic structures which are thought to play pivotal roles in their mobilization and dissemination (Poirel and Nordmann, 2006; Zarrilli et al., 2008; D'Andrea et al., 2009; Merino et al., 2010; Poirel et al., 2010; Ravasi et al., 2011; Towner et al., 2011; Grosso et al., 2012; Roca et al., 2012; Evans and Amyes, 2014; Fu et al., 2014; Nigro et al., 2015; Da Silva and Domingues, 2016). A detailed characterization of the plasmids carried by carbapenem-resistant *A. baumannii* strains may help our understanding of the mechanisms of dissemination of these resistance structures, and contribute to the adoption of measures that limit the spread of antimicrobial resistance at both local and global scales.

Several authors have noted that many plasmid-borne *bla*_OXA_-containing structures are bordered by short sequences displaying homology to the 28-nucleotide *dif* motif located at the bacterial chromosome replication terminus and recognized by the XerC/D site-specific recombinases, leading to proposals that their mobilization could be mediated by site-specific recombination (Poirel and Nordmann, 2006; Zarrilli et al., 2008; D'Andrea et al., 2009; Merino et al., 2010; Poirel et al., 2010; Towner et al., 2011; Grosso et al., 2012; Evans and Amyes, 2014; Fu et al., 2014; Da Silva and Domingues, 2016; Blackwell and Hall, 2017). The highly conserved Xer site-specific recombination system normally acts to resolve bacterial chromosome dimers that form by homologous recombination during DNA replication, therefore allowing normal chromosome segregation to daughter cells during cell division (Cornet et al., 1994; Carnoy and Roten, 2009; Tran et al., 2012; Colloms, 2013; Midonet and Barre, 2014; Castillo et al., 2017). This resolution is mediated by the XerC and the XerD tyrosine recombinases which act coordinately to catalyze the addition of a cross-over between a directly-oriented pair of *dif* sites. Many plasmids contain sites recognized by the XerC/D recombinases (XerC/D-like sites) and exploit the host Xer system to resolve their own multimeric states, thus avoiding segregational instability or “dimer catastrophe” (Cornet et al., 1994; Carnoy and Roten, 2009; Garcillán-Barcia et al., 2011; Tran et al., 2012; Colloms, 2013; Midonet and Barre, 2014; Castillo et al., 2017). In addition, a number of mobile elements collectively designated as integrating mobile element exploiting Xer (IMEX) use this system to integrate (and eventually excise) their genomes into (from) the chromosomes of their respective hosts (Midonet and Barre, 2014; Castillo et al., 2017). Studies with model systems have indicated that, depending on their particular sequences and the immediate genetic context in which they are embedded, a pair of XerC/D-like sites may behave differently in recombination (Cornet et al., 1994; Colloms, 2013). Thus, a given pair may mediate only intra-molecular resolution, allow both intra-molecular resolution and inter-molecular fusion, or be inactive (Cornet et al., 1994). In this context whether the XerC/D-like sites linked to *bla*_OXA_-containing structures carried by *A. baumannii* plasmids are active, how do they act in recombination, and how this behavior may influence the mobilization and eventual dissemination of these resistance structures is still poorly understood.

We previously reported the presence of a plasmid-borne *bla*_OXA-58_ gene overexpressed as the result of the upstream insertion of an IS*Aba825* element among a group of clonally- and epidemiologically-related MDR *A. baumannii* clinical strains isolated in public hospitals of Rosario, Argentina (Ravasi et al., 2011; Piazza et al., 2013). In this work we characterized in detail the plasmids present in one of these strains, designated Ab242, to obtain clues into their dynamics and roles in the dissemination of carbapenem resistance determinants among the *Acinetobacter* clinical population.

Part of these results was presented recently in the 11th International Symposium on the Biology of *Acinetobacter*, Seville, Spain (Cameranesi et al., 2017a).

## Materials and methods

### *A. baumannii* strains employed in this work and growth conditions

*Acinetobacter baumannii* Ab242 is a MDR clinical strain displaying carbapenem resistance (Table S1) isolated in 1997 from a clonal group disseminating in a public healthcare institution of Rosario, Argentina (Limansky et al., 2004; Mussi et al., 2011). MLST analysis assigned this strain to ST104 (CC104) in the Oxford scheme (*cpn60*-29, *gdhB*-12, *gltA*-12, *gpi*-57, *gyrB*-17, *recA*-1, *rpoD*-39) and to ST15 in the Pasteur scheme (*cpn60*-6, *fusA*-6, *gltA*-8, *pyrG*-2, *recA*-3, *rplB*-5, *rpoB*-4) (https://pubmlst.org/bigsdb?db=pubmlst_abaumannii_oxford_seqdef).

The *A. nosocomialis* M2 strain (Carruthers et al., 2013) was used in this study as a recipient for transformation assays with plasmids extracted from Ab242 (see below).

The above *Acinetobacter* strains were routinely grown in Lysogeny Broth (LB) liquid medium at 37°C under aerobic conditions with vigorous shaking for plasmid extraction procedures, or in LB agar medium with the indicated antibiotics for transformation studies and the selection of individual colonies. Appropriate procedures for working with a level 2 pathogen were followed throughout this work (Biosafety in Microbiological and Biomedical Laboratories, 5th edition, U.S. Department of Health and Human Services, Centers for Disease Control and Prevention, National Institutes for Health).

### Antimicrobial susceptibility assays

The MICs for different antimicrobials of *A. baumannii* Ab242, *A. nosocomialis* M2, and *A. nosocomialis* M2 transformed with Ab242 plasmids (Table S1) were evaluated using the VITEK-2 antimicrobial susceptibility testing system (bioMérieux). The MICs for kanamycin were determined by the macrodilution method using Mueller-Hinton (MH) broth in accordance to CLSI recommended procedures (Clinical and Laboratory Standards Institute, 2016).

### Plasmid isolation and analysis

The plasmids from Ab242 or from transformed *A. nosocomialis* cells were extracted using the Wizard® DNA purification kit (Promega, Madison, WI, USA) or the E.Z.N.A® Plasmid DNA Mini Kit I (OMEGA bio-tek, Norcross, GA, USA). The plasmids were analyzed by 0.7% agarose gel electrophoresis and ethidium bromide staining following conventional procedures (Sambrook et al., 1989).

### Transformation of *A. nosocomialis* M2 cells with Ab242 plasmids

Ab242 plasmids were transformed into competent cells of *A. nosocomialis* M2 by electroporation using a Bio-Rad GenePulser II set at 2.5 kV, 25 mF, and 200 V. The transformed cell mixture was plated on LB agar supplemented with 2 μg/ml IPM incubated overnight at 37°C and resistant colonies were analyzed for the presence of plasmids bearing the IS*Aba825-bla*_OXA-58_ arrangement by PCR using primers P*ISAba825*-F and OXA-58R (Table S2).

Plasmids extracted from the transformed *A. nosocomialis* cells were first characterized by restriction mapping with EcoRI and BamHI. The EcoRI-derived fragments were further cloned into *E. coli* cloning vectors for sequencing purposes. Briefly, these fragments were ligated into the equivalent sites of the pSU18 plasmid bearing a chloramphenicol (Cm)-resistance cassette (Bartolome et al., 1991), transformed into *E. coli* DH5α competent cells, and plated on LB agar medium containing 20 μg/ml Cm, 1 mM IPTG, and 0.5 mM X-gal to identify *E. coli* colonies bearing insert-containing plasmids (Bartolome et al., 1991). These plasmids were further isolated using the Wizard DNA purification kit (Promega, Madison, WI), restriction mapped with EcoRI, and selected inserts were further subjected to DNA sequencing (see below).

### S1 nuclease and southern blot analysis of *acinetobacter* plasmids

**Figure 1:**
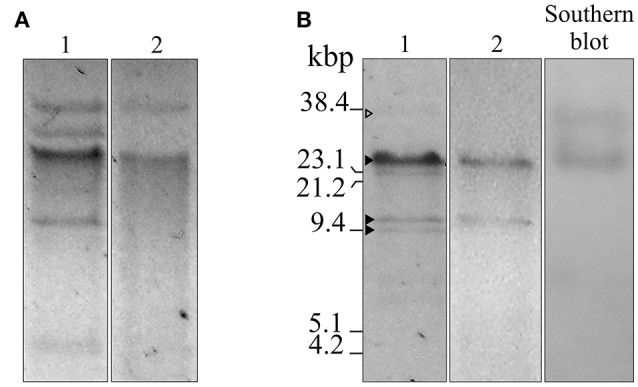
Agarose gel electrophoresis, S1 nuclease, and Southern blot analyses of plasmids isolated from *A. baumannii* Ab242 and *A. nosocomialis* M2 transformed with Ab242 plasmids. **(A)** Gel electrophoresis (0.7% agarose)/ethidium bromide staining of total plasmids extracted from Ab242 cells (lane 1) and *A. nosocomialis* M2 cells transformed with Ab242 plasmids (lane 2). **(B)** Ab242 total plasmids were treated with S1 nuclease (lane 1) and plasmids extracted from *A. nosocomialis* M2 cells transformants were treated with BamHI (lane 2), both analyzed by agarose gel electrophoresis/ethidium bromide staining. The gels from lane 1 were capillary transferred to nitrocellulose membranes and subjected to a Southern blot analysis using a 689-bp biotin-labeled *bla*_OXA-58_ probe (lane 3). The three closed arrowheads at the left margin indicate, from top to bottom, discrete DNA bands obtained after S1 nuclease digestion of sizes of around 25, 12, and 9 kbp, respectively. The open arrowhead at the top points to faint band of a larger size. The final positions of the size markers (38.4 kbp SacI-digested pLD209 plasmid, Marchiaro et al., 2014; plus lambda DNA digested with EcoRI and HindIII) are indicated at the left margin.

S1 nuclease treatment of Ab242 plasmids was conducted essentially as described (Barton et al., 1995) followed by agarose gel electrophoresis analysis of the digested material (Figure 1B). Southern blot analysis was performed on these gels following described protocols (Sambrook et al., 1989). Briefly, the gels were incubated in 0.25 M HCl for 10 min, then in 0.5 M NaOH, 1.5 M NaCl for 20 min, and finally equilibrated in 1 M Tris-HCl (pH 7.0), 1.5 M NaCl, and pressure blotted for 16 h into a nitrocellulose membrane (Amersham Biosciences Hybond™ -ECL, GE Healthcare UK Limited, UK). The membranes were hybridized with a biotinilated probe specific for the *bla*_OXA-58_ gene made of a 689-bp PCR-generated fragment using biotin-labeled primers (OXA-58-Fw-5′Biot and OXA-58-Rv-5′Biot, see Table S2; Sambrook et al., 1989). After incubation with a streptavidine-alkaline phosphatase conjugate (Sigma-Aldrich®, Saint Louis, MI, USA), specific bands were detected by chromogenic blot development with nitro-blue tetrazolium and 5-bromo-4-chloro-3′-indolyphosphate.

### Plasmid sequencing, assembly, and comparative sequence analyses

DNA sequencing of Ab242 plasmids was done at the Instituto de Agrobiotecnología Rosario (INDEAR, Rosario, Argentina) using a 454 pyrosequencing platform (Roche Diagnostics). The obtained reads were assembled *in silico* and the resulting sequences were refined by visual inspection. Three plasmids were inferred from this analysis and designated pAb242_25, pAb242_12, and pAb242_9, where the latter numbers indicate the approximate corresponding lengths in kbp. The circular structures of these plasmids were confirmed by PCR using specifically designed primer pairs followed by sequencing of the obtained amplicons, as well as by primer walking (see Table S2 and Figure S1 for details). DNA sequencing was done at the Sequencing Facility of Maine University. The nucleotide sequences of the plasmids described in this study were deposited in the GenBank nucleotide sequence database under accession numbers KY984047 (pAb242_25), KY984046 (pAb242_12), and KY984045 (pAb242_9).

The Rapid Annotation using Subsystem Technology standard operating procedures (RAST, http://rast.nmpdr.org/seedviewer.cgi) (Aziz et al., 2008) and the National Center for Biotechnology Information database (NCBI, U.S. National Library of Medicine, Bethesda MD, USA) were used to annotate the open reading frames (ORFs).

Search for antimicrobial resistance determinants was done using ResFinder 2.1 (https://cge.cbs.dtu.dk/services/ResFinder/; Zankari et al., 2012). The detection of IS was done with IS Finder (Siguier et al., 2006) (https://www-is.biotoul.fr/) and ISsaga (Varani et al., 2011). Comparative analysis of the carbapenem and aminoglycoside adaptive module located in pAb242_25 with similar structures reported in other *Acinetobacter* plasmids was conducted using Mauve (Darling et al., 2010).

### Definition of a XerC/D-like recognition motif and detection of equivalent sites in Ab242 plasmids

A consensus 28-mer XerC/D recognition sequence (5′-atTTcgtaTAAggtgtaTTATGttAAat-3′) was generated from the comparisons of 17 reported *Acinetobacter* XerC/D-like sites, which included the single *dif* site present in the *Acinetobacter baylyi* chromosome (Carnoy and Roten, 2009) and 16 sites described in different *Acinetobacter* plasmids (Table S5). In the above consensus sequence the uppercase letters denote a completely conserved nucleotide at a given position in the 17 sequences analyzed, otherwise lowercase letters were used. The “*Consensus finder*” online tool (http://www.insilicase.com/Web/ConsensusSite.aspx) was used employing the above motif and allowing for up to two mismatches at conserved positions for the detection of XerC/D-like sites in Ab242 plasmids.

## Results

### Plasmids harbored by Ab242

The *A. baumannii* Ab242 clinical strain used in this study was assigned to the ST104 of the CC104 (Oxford scheme), one of the CCs responsible for the dissemination of *bla*_OXA-58_ genes in South America (Clímaco et al., 2013; Ramírez et al., 2013). Antimicrobial susceptibility testing indicated a MDR profile for Ab242, with clinical resistance to β-lactams including carbapenems (IPM, meropenem), extended spectrum cephalosporins (cefotaxime, ceftazidime, cefepime), ampicillin/sulbactam and piperacillin/tazobactam; as well as to aminoglycosides (amikacin, gentamicin); to quinolones (ciprofloxacin); and to folate pathway inhibitors (trimethoprim/sulphamethoxazole) (Table S1).

**Figure 2:**
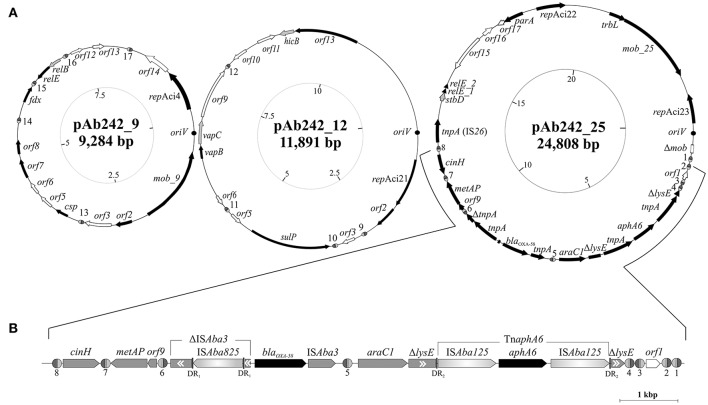
Organization and gene content of *A. baumannii* Ab242 plasmids. **(A)** From left to right: inferred schemes of Ab242 plasmids pAb242_9 (KY984045), pAb242_12 (KY984046), and pAb242_25 (KY984047). The ORFs and their corresponding orientations are denoted by arrows. Closed arrows correspond to genes with described functions, whereas open arrows relate to unknown functions. Disrupted and incomplete genes are indicated with a “Δ” symbol preceding the gene denomination. The dark circles denote the predicted *oriV* regions, whose starting bases were arbitrarily set to 1 in the clockwise scale in each case (innermost circles) that also indicates the corresponding lengths in kbp. The different XerC/D-like binding sites identified in this work are highlighthed as ovals (not drawn to scale), with the XerC and XerD recognition regions depicted as dark gray and light gray semi-ovals, respectively, separated by the corresponding cr. Note that not all sites located in a given plasmid show the same polarity. The sequences of the different XerC/D sites are shown in Table **2**. For more details see also Table S3. **(B)** Expanded view of the antimicrobial resistance structure bearing *bla*_OXA-58_ and *aphA6* antimicrobial resistance genes and its close bordering regions. The orientation of each gene is indicated, with the two resistance genes denoted in black. Genes interrupted by IS are indicated in gray boxes with a “Δ” symbol, with the double internal arrowheads indicating the original direction of transcription. Thus, ΔIS*Aba3* located immediately upstream of *bla*_OXA-58_ denotes the remnants of an original IS*Aba3* copy disrupted by an IS*Aba825* insertion. In turn, the two Δ*lysE* fragments bordering the Tn*aphA6* composite transposon denote the 5′ and 3′ remnants, respectively, of the original *lysE* gene. DR_1_ and DR_2_ denote the direct duplications generated by the indicated mobile elements at the corresponding target insertion sites (see Table S4 for details).

Plasmid extraction followed by agarose gel electrophoresis analysis indicated the presence of plasmids in Ab242 (Figure 1A). S1 nuclease analysis revealed the linearized plasmid forms showing at least 3 well-defined species whose sizes ranged from around 9 kbp to slightly more than 23 kbp, from which the latter appeared as the predominant plasmid form (Figure 1B). Pyrosequencing analysis followed by *in silico* assembly and gap closure (Figure S1A, Table S2, see Materials and Methods for details) indicated three plasmids in this strain of approximate sizes of 9, 12, and 25 kbp hereafter designated pAb242_9, pAb242_12, and pAb242_25, respectively (Figure 2A). Database sequence comparisons predicted the presence of 15 complete ORFs in pAb242_9 from which 9 encode proteins with attributed functions (black arrows in Figure 2A); 13 complete ORFs in pAb242_12 from which 6 encode proteins with attributed functions; and 21 complete ORFs in pAb242_25 from which 16 encode proteins with attributed functions (see Table S3 for details). From them only pAb242_25 carried antimicrobial resistance determinants, including a *bla*_OXA-58_ gene preceded by an IS*Aba825* insertion promoting overexpression of the CHDL gene (Ravasi et al., 2011) accompanied by a composite transposon (Tn*aphA6*) bearing an *aphA6* aminoglycoside resistance gene (Nigro et al., 2011). Transformation analyses of susceptible *Acinetobacter* strains indicated that the above genes conform an antimicrobial resistance module effectively conferring both carbapenem- and aminoglycoside-resistance to an *Acinetobacter* host (Table S1, see also below). Of note also, pAb242_25 was the only plasmid identified in Ab242 carrying different IS including IS*26*, IS*Aba825*, IS*Aba125*, and IS*Aba3* (Iida et al., 1984; Mussi et al., 2005; Poirel and Nordmann, 2006; Ravasi et al., 2011; Figure 2A, Tables S3, S4).

### Replication, stability, transferability, and adaptive modules in Ab242 plasmids

The replication modules of *Acinetobacter* plasmids differ from those of other bacterial groups, leading to a classification scheme based on the comparison of replicase (Rep) sequences that originally distinguished 19 different homology groups (GR) among them (Bertini et al., 2010; Towner et al., 2011). Our searching predicted a total of four *rep* genes among Ab242 plasmids, all encoding replicases of the Rep-3 superfamily (Table 1; Bertini et al., 2010; Towner et al., 2011). A more detailed characterization of the encoded proteins against representative replicases of each of the presently defined *A. baumannii* plasmids GR groups confidentially assigned the replicase encoded in pAb242_9 to GR4, as judged by the 95% sequence identity with the RepAci4 enzyme from *A. baumannii* p844 (Table 1, Table S3). On the contrary, the affiliation of the three other Ab242 plasmids replicases to any of the 19 presently-defined GR was more ambiguous. For instance, in the bi-replicon plasmid pAb242_25 (Figure 2, Table S3) one of the *rep* genes (designated *rep*Aci23, Table 1) encodes a protein with 100% amino acid identity with the Rep protein encoded in plasmid p11921 (Table S3), which was marginally affiliated to GR8 in the current classification scheme (Bertini et al., 2010). Also, the closest assigned homolog to the second replicase encoded in pAb242_25 (designated *rep*Aci22, Table 1) was the RepAciX protein (GR10) from pACICU1, but with only 53% identity at the protein sequence level, thus uncovering a novel class of replicase in pAb242_25. Similarly, the closest assigned homolog of the replicase encoded in pAb242_12 (designated *rep*Aci21, Table 1) was the GR12 enzyme from pABIR, but again with only 41% protein identity. These comparisons suggested the presence of three novel replicases in Ab242 plasmids. In this context, we have recently re-evaluated the Rep-based strategy of *A. baumannii* plasmids classification using 122 replicases sequences deposited in public databases. This analysis confirmed that *rep*Aci21, *rep*Aci22, and *rep*Aci23 represent examples of three novel GR groups (Cameranesi et al., 2017b).

**Table 1:** Stability, propagation and adaptive modules in Ab242 plasmids.

**Module**	**Plasmid**	**CDS**	**Description**	**Comments**	**GenBank accession number**
Replication	pAb242_9	*rep*Aci4	Replicase, GR4	Rep-3 superfamily (pfam01051)	KY984045
	pAb242_12	*rep*Aci21	Replicase, GR21^a^	Rep-3 superfamily (pfam01051)	KY984046
	pAb242_25	*rep*Aci22	Replicase, GR22^a^	Rep-3 superfamily (pfam01051)	KY984047
		*rep*Aci23	Replicase, GR23^a^	Rep-3 superfamily (pfam01051)	
Stability	pAb242_9	*relE*	RelE toxin of type II RelBE TA system	ParE toxin superfamily (pfam05016)	KY984045
		*relB*	RelB antitoxin of RelBE TA system	RelB superfamily (pfam04221)	
	pAb242_12	*vapC*	VapC toxin of type II VapCB TA system	PIN domain (pfam01850)	KY984046
		*vapB*	VapB antitoxin of VapCB TA system	MazE_antitoxin (pfam04014)	
		*orf13*	Putative HicA toxin of HicAB TA system	Not detected	
		*hicB*	Putative HicB antitoxin of HicAB TA system	Not detected	
	pAb242_25	*relE_1*/*relE_2*^b^	RelE toxin of type II RelE/StbD TA system, splitted	ParE toxin superfamily (pfam05016)	KY984047
		*stbD*	StbD antitoxin of RelE/StbD TA system	Phd_YefM superfamily (pfam02604)	
Mobility	pAb242_9	*mob_9*	Nickase/mobilization protein	MobA/MobL (pfam03389)	KY984045
	pAb242_25	*mob_25*	Relaxase mobilization protein	Relaxase/Mobilization nuclease domain (pfam03432)	KY984047
		*trbL*	Conjugal transfer protein (MobC)	Bacterial mobilization protein MobC (pfam05713)	
Adaptive	pAb242_25	*bla*_OXA-58_	Genetic structure containing *bla*_OXA-58_ and *aphA6* genes conferring resistance to carbapenems and aminoglycosides	β-lactamase class D (COG2602)	KY984047
		*aphA6*		Aminoglycoside phosphotransferase (COG3231)	

a *Replicase assigned to a novel homology group (GR), see text for explanation*.

b *The relE gene contains a mutation introducing a premature termination codon after amino acid 34 in the coding sequence (see Table S3 for details)*.

Concerning stability function genes, different toxin-antitoxin (TA) systems (Bertini et al., 2010; Fondi et al., 2010; Garcillán-Barcia et al., 2011; Towner et al., 2011) were detected in Ab242 plasmids (Figure 2A, Table 1 and Table S3). Each of the Ab242 plasmids carries at least one TA system which include RelBE-type and VapBC-type systems (Table 1). Homologous systems were separately detected in other *Acinetobacter* plasmids including pAB2, pM131-5, and pACICU1 (Jurėnaitė et al., 2013), which otherwise show little sequence homology to Ab242 plasmids (Figure S2). This strongly suggests that these TA systems are mobilized as modules between the *Acinetobacter* population. It is worth noting that the *relE* toxin gene of the TA module located in pAb242_25 contains a mutation introducing a premature termination codon splitting this gene in two regions (Table 1, Table S3). This premature interruption was not observed in the *relE* gene of the homologous and active TA system present in pACICU1 (Jurėnaitė et al., 2013), casting doubts on the functionality of the RelE toxin in pAb242_25.

Regarding transferability genes no complete conjugative systems were detected among Ab242 plasmids, in line with previous observations indicating that most *A. baumannii* plasmids generally lack transfer functions (Bertini et al., 2010; Fondi et al., 2010; Garcillán-Barcia et al., 2011; Towner et al., 2011). Still, some *mob* homologs were detected in pAb242_9 and pAb242_25 such as *mob_9* and the cluster *mob_25*-*trbL*, respectively (Table 1, Table S3).

Concerning antimicrobial resistance genes, as noted above only pAb242_25 carries an adaptive module of around 7.8 kbp containing both *bla*_OXA-58_ and *aphA6* genes in which different IS play roles in the overexpression (IS*Aba825*, Ravasi et al., 2011) or in the mobilization (IS*Aba3*, Poirel and Nordmann, 2006; IS*Aba125*, Lambert et al., 1990; Nigro et al., 2011) of these resistance genes (Figure 2B, Table 1, Tables S3, S4). The *bla*_OXA-58_ gene is immediately flanked by the remnants of a composite transposon originally formed by two oppositely-oriented IS*Aba3* copies (Figure 2B) as in other *Acinetobacter* plasmids carrying related structures (Poirel and Nordmann, 2006; Zarrilli et al., 2008; D'Andrea et al., 2009; Evans and Amyes, 2014). The insertion of IS*Aba825* at the IS*Aba3* copy located immediately upstream of the *bla*_OXA-58_ gene (Figure 2B) has been found to generate an alternative strong promoter driving overexpression of the CHDL gene (Ravasi et al., 2011). Similarly to related structures (Poirel and Nordmann, 2006; Zarrilli et al., 2008; D'Andrea et al., 2009; Ravasi et al., 2011; Evans and Amyes, 2014; Fu et al., 2014), an *araC1* gene was located downstream of the intact IS*Aba3* copy of the composite transposon, followed in turn by a *lysE* gene disrupted in this case by a Tn*aphA6* formed by two IS*Aba125* elements bracketing an *aphA6* gene (Nigro et al., 2011). Tn*aphA6* and IS*Aba825* likely represent recent acquisitions by this adaptive module as judged by the noticeable retention of the short direct duplications generated by the insertions of these mobile elements at the corresponding target sites (Figure 2B and Table S4).

### XerC/D-like sites in Ab242 plasmids

Short DNA stretches displaying homology to the recognition motif of XerC and XerD site-specific recombinases (designated Re27, XerC/D-like, or p*dif* sites by different authors) have been previously noticed at the borders of *bla*_OXA-58_-containing resistance structures carried by *Acinetobacter* plasmids (Poirel and Nordmann, 2006; Zarrilli et al., 2008; D'Andrea et al., 2009; Merino et al., 2010; Towner et al., 2011; Grosso et al., 2012; Evans and Amyes, 2014; Fu et al., 2014; Da Silva and Domingues, 2016; Blackwell and Hall, 2017). We obtained a 28-mer consensus motif from the comparisons of XerC/D-like recognition sites described in *Acinetobacter* plasmids (Table S5), and used it as a query to detect similar sequences among Ab242 plasmids (see Materials and Methods for details). Our searching identified 17 XerC/D-like sites among them: 5 in pAb242_9; 4 in pAb242_12; and 8 in pAb242_25 (Table 2). As seen in Table 2, the XerC/D-like sites identified in Ab242 plasmids were not identical between them, with the XerD recognition region more conserved than the corresponding XerC region and the central region (cr) being the most variable, similarly to the situation of chromosomal *dif* sites (Carnoy and Roten, 2009). Also reflecting the case of chromosomal *dif* sites, the XerC/D sites identified in Ab242 plasmids possess the highly conserved positions 8 to 11 (XerC recognition region) and 18 to 21 (XerD recognition region) bordering the cr (Table 2) which are critical for the binding of the corresponding recombinases (Carnoy and Roten, 2009).

**Table 2:** XerC/D-like recognition sites found in Ab242 plasmids.

**Plasmid**	**XerC/D sites^a^**	**Nucleotide sequence^b^**			**Position in plasmid (bp)**
	**XerC/D motif**	**XerC**	**cr**	**XerD**	
pAb242_25	XerC/D_1	·c·g··c····	· aga· **t**	···········	1,312–1,339
	XerC/D_2	······c····	····· ·	········· t ·	1,367–1,394
	XerC/D_3	··· gt ······	**caacc**·	·········g ·	1,832–1,859
	XerC/D_4	·· ·········	**cagcc**·	···········	1,936–1,963
	XerC/D_5	····t ······	····· ·	········· t ·	6,578–6,605
	XerC/D_6	**g** ········· ·	**cagcc**·	····c······	9,845–9,872
	XerC/D_7	··· a······ ·	····· ·	········· t ·	10,925–10,952
	XerC/D_8	· a···t···· ·	· aaa· **t**	······g·· t·	11,831–11,858
pAb242_12	XerC/D_9	···· ·······	····· ·	········ tt ·	2,263–2,290
	XerC/D_10	· c········ ·	····· ·	········· t ·	2,802–2,829
	XerC/D_11	**g** ········· ·	**c**·ccc ·	···········	4,935–4,962
	XerC/D_12	······c····	· aga· **t**	········· ·**c**	7,183–7,210
pAb242_9	XerC/D_13	······c····	· aga· **t**	········· ·**g**	2,702–2,729
	XerC/D_14	· ··········	**c**·ccc ·	···········	5,057–5,084
	XerC/D_15	**cc** ·········	····· ·	···········	5,739–5,766
	XerC/D_16	·c···· c····	**tcgcc** ·	···········	6,384–6,411
	XerC/D_17	· ··········	**c**·····	········· t ·	7,261–7,288
pAb242_37^c^	XerC_9/XerD_7	···· ·······	····· ·	········· t ·	–
	XerC_7/XerD_9	··· a······ ·	····· ·	········ tt ·	–
	Ab242 Consensus^d^	**atTtcgtATAA**	**ggtgta**	**TTATgTtAaat**	

a *The numbers assigned to the different XerC/D-like recognition sites located in Ab242 plasmids were arbitrarily chosen*.

b *In each of the inferred XerC/D sites the presence of the same nucleotide in a given position (reflecting an invariant positions in all 17 sequences) is denoted with a dot sign (.), otherwise the corresponding nucleotide is indicated in lowercase letters*.

c *Plasmid resulting from the fusion of pAb242_25 and pAb242_12*.

d *New consensus derived from the above XerC/D-like sites identified in Ab242 plasmids*.

Of note, Ab242 plasmids are endowed with 3.7 XerC/D-like sites per 10 kbp of length in average, a number far exceeding the single site found on whole bacterial chromosomes, on most other plasmids using the Xer system for multimer resolution, or on IMEX elements (Cornet et al., 1994; Carnoy and Roten, 2009; Tran et al., 2012; Colloms, 2013; Midonet and Barre, 2014; Castillo et al., 2017). Moreover, since the different XerC/D sites are neither palindromic nor identical (e.g., see Table 2), they show defined polarities when located in the same plasmid molecule with some displaying the same orientation (directly-oriented sites) and others opposite orientations (inversely-oriented sites) (Figure 2A). Since the outcome of intra-molecular recombinational exchanges occurring on a circular molecule differs depending on the relative orientations of the recombining sister sites (Colloms, 2013), both the content and varied orientations of XerC/D-like sites in each of the Ab242 plasmids (Figure 2A) suggest function(s) other than a sole participation in the resolution of the corresponding multimeric forms as is other cases.

In the above context we noted that 7 out of the 8 XerC/D-like sites identified in pAb242_25 are located in the close vicinity of the adaptive module carried by this plasmid, while the remaining site (#5) is embedded between the intact IS*Aba3* copy located downstream of *bla*_OXA-58_, and the *araC1* gene (Figure 2B). Three XerC/D-like sites designated from #6 to #8 (Table 2) were located upstream of the *bla*_OXA-58_ gene (left side of the adaptive module in Figure 2B), with site #6 positioned closer to the disrupted IS*Aba3* copy. Site #7 is separated from #6 by a *metAP* gene and an *orf9*, and displays an inverse-orientation as compared to #6. In turn, site #8 is separated from #7 by a *cinH* gene, and is directly-oriented as compared to #7 (Figure 2B). On the opposite extreme of the adaptive module four XerC/D-like sites were located and designated from #4 to #1, with site #4 situated immediately downstream of the 3′end of the disrupted *lysE* gene and directly-oriented as compared to site #6 (Figure 2B). Notably, sites #4, #3, and #2 are positioned in alternate inverse orientations between them, while #1 shows the same orientation as site #2.

### A pair of XerC/D sister sites allowing reversible co-integrate formation between plasmids pAb242_25 and pAb242_12

We analyzed next whether the adaptive module present in pAb242_25 could induce simultaneous resistance to carbapenems and aminoglycosides when introduced into a susceptible *Acinetobacter* host. For this purpose total plasmids were extracted from Ab242 and electrotransformed into susceptible *A. nosocomialis* M2 cells (see Materials and Methods for details). The transformed cells were plated on LB agar supplemented with 2 μg/ml IPM to select for carbapenem resistance, and after incubation different colonies were screened for the presence of the IS*Aba825*-*bla*_OXA-58_ arrangement by PCR. The antimicrobial susceptibility profile of a representative *A. nosocomialis* transformant clone testing positive for this arrangement is shown in Table S1. As seen in this Table, the corresponding cells had acquired resistance not only to carbapenems such as IPM and meropenem but also to several aminoglycosides including amikacin and kanamycin, thus indicating the presence of the adaptive module containing the IS*Aba825*-*bla*_OXA-58_ arrangement and the Tn*aphA6* transposon.

**Figure 3:**
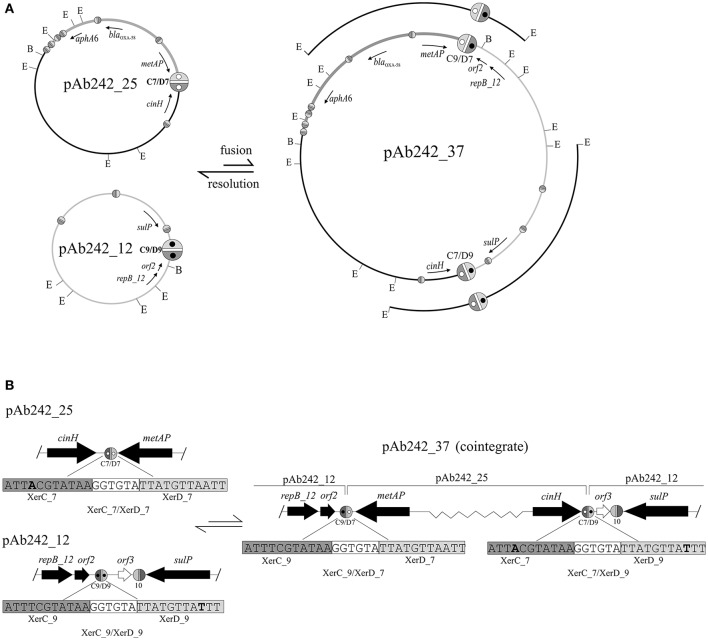
Site-specific recombination between XerC/D sites mediates fusion between pAb242_25 and pAb242_12. (**A**, Upper panel) Scheme depicting the formation of the plasmid co-integrate pAb242_37 by inter-molecular site-specific recombination at the sister pair XerC_7/XerD_7 (C7/D7) and XerC_9/XerD_9 (C9/D9) located in pAb242_25 and pAb242_12, respectively. The resolution of pAb242_37 by intra-molecular site-specific recombination at the new pair of XerC/D sites generated during the fusion reaction (C9/D7 and C7/D9, respectively) is also shown. The *bla*_OXA-58_- and *aphA6*-containing adaptive module in pAb242_25 and pAb242_37 is highlighted in dark gray. The positions of EcoRI (E) and BamHI (B) restriction sites in these plasmids are also indicated. The different XerC/D-like recognition sites (see Table 2 for sequence details) are highlighted as ovals (not drawn to scale) as detailed in the legend to Figure 2. The pair of sites identified as recombinationally active in this work have been arbitrarily enlarged. Open and closed inner circles within XerC_7/XerD_7 and XerC_9/XerD_9, respectively, are shown to facilitate the visualization of the fusions generating XerC_9/XerD_7 (C9/D7) and XerC_7/XerD_9 (C7/D9) in pAb242_37. The cloned EcoRI fragments used to identify these fusions (see text for details) are also indicated above the pAb242_37 scheme. (**B**, Lower panel) Enlarged vision including the nucleotide sequences of the XerC/D sites involved in the fusion/resolution reactions showing that the polarity of the sites was maintained after site-specific recombination. Relevant nucleotides in each site are highlighted in bold. Slash (/) symbols at the borders indicate that further sequence data existed beyond this position. The scheme is not drawn to scale.

A more detailed characterization of the plasmids extracted from the transformed *A. nosocomialis* cells was then conducted. The plasmid pattern obtained after agarose gel electrophoresis analysis showed an expected lower number of bands as compared to that generated by Ab242 cells (Figure 1A). However, a BamHI restriction enzyme analysis (lane 2 in Figure 1B) revealed that the (expected) large fragment of approximately 25 kbp corresponding to the BamHI-linearized form of pAb242_25 was accompanied by an extra band of around 12 kbp (for the location of BamHI sites in pAb242_25 and pAb242_12 see Figure 3A). As seen in Figure 3A the extra 12 kbp band corresponds to the BamHI-linearized form of pAb242_12, suggesting that the plasmid originally transforming and establishing in *A. nosocomialis* was a co-integrate between pAb242_25 and pAb242_12 which had then undergone resolution in this new host (see scheme in Figure 3A).

At least two different mechanisms, one involving transposition of a mobile element (Iida et al., 1984; Mahillon and Chandler, 1998) and the other a fusion reaction mediated by site-specific recombination at XerC/D sites (Cornet et al., 1994; Tran et al., 2012; Colloms, 2013; Midonet and Barre, 2014; Castillo et al., 2017) could account for the formation of co-integrates between two circular DNA molecules lacking shared long regions of sequence identity. If co-integrate formation between pAb242_25 and pAb242_12 resulted from recombinational exchange between a sister pair of XerC/D sites, the polarity of the recombining sites would be maintained so that, after cleavage by the recombinases, the left-hand half of each site would be rejoined to the right-hand half of the other on the fused product (Carnoy and Roten, 2009; Tran et al., 2012; Colloms, 2013; Midonet and Barre, 2014; Castillo et al., 2017). In turn, if co-integrate formation resulted from the transposition of a mobile element originally present in one of the plasmids, the responsible element would be retained as a scar in the fusion site linking both plasmids (Iida et al., 1984; Mahillon and Chandler, 1998). Thus, the characterization of the fusion region between the two plasmids can allow differentiating between the two potential mechanisms of co-integrate formation.

We thus attempted the identification of enzyme restriction fragments containing fusions between pAb242_25 and pAb242_12 in the plasmid mixture obtained from the *A. nosocomialis* transformant described above. The plasmid mixture was digested with EcoRI and the derived fragments were cloned into the equivalent sites of the *E. coli* vector plasmid pSU18 and analyzed (see Materials and Methods for details). From more than 100 inserts analyzed we identified two EcoRI fragments of around 8 kbp and 11 kbp, respectively, containing the products corresponding to a fusion between sites XerC_9/XerD_9 of pAb242_12 and XerC_7/XerD_7 of pAb242_25, i.e., XerC_9/XerD_7 and XerC_7/XerD_9 (Figure 3 and Table 2 for sequence details). The identification of this particular fusion confirmed the formation of a co-integrate between pAb242_12 and pAb242_25 (i.e., pAb242_37) from an inter-molecular site-specific recombinational exchange mediated by the indicated pair of XerC/D sites.

The presence of pAb242_37 in the plasmid mixture recovered from the *A. nosocomialis* transformant cells was subsequently confirmed using a combination of PCR and primer walking strategies specifically designed to identify XerC_9/XerD_7 and XerC_7/XerD_9 fusions (Figure S1B). By using similar procedures (Figure S1A) we identified also the presence of plasmids bearing the original XerC_7/D_7 and XerC_9/XerD_9 sites in this mixture, in agreement to our results above indicating that pAb242_37 had mostly undergone resolution in the *A. nosocomialis* transformant cells (Figure 3A). Of note, the two pairs of sister sites identified above share a common cr sequence, GGTGTA, but show one-nucleotide differences at both the XerC (position 4) and XerD (position 26) recognition motifs (Table 2). Still, these variant positions are relatively separated from the conserved palindromic sequence encompassing positions 8 to 11 and 18 to 21 bracketing cr and presumed critical for the binding of the recombinases for subsequent cleavage (Carnoy and Roten, 2009).

Similar PCR and primer walking strategies described above (Figure S1B) were also used to verify the presence of the pAb242_37 co-integrate in plasmid extracts obtained from Ab242 cells. An evaluation of the relative abundance of this co-integrate as compared to pAb242_25 was then attempted on S1 endonuclease digests by means of a Southern blot analysis using *bla*_OXA-58_ as a probe (Figure 1B). As seen in this Figure, the analysis indicated the presence of two forms carrying *bla*_OXA-58_, a main band of approximately 25 kbp corresponding to the linearized form of pAb242_25 and a less prominent band of a larger size most likely representing the pAb242_37 co-integrate.

The overall results shown above support the existence on Ab242 cells of a co-integrate resulting from the fusion of pAb242_25 and pAb242_12 mediated by an inter-molecular site-specific recombination involving sites XerC/D_7 in pAb242_25 and XerC/D_9 in pAb242_12. Moreover, they also indicated that the two XerC/D sites product of this recombinational exchange provided another active sister pair, thus allowing co-integrate resolution (see scheme in Figure 3). The low recovery of restriction fragments containing fused products between pAb242_25 and pAb242_12 in the plasmid mixture derived from transformed *A. nosocomialis* cells, added to the higher abundance of pAb242_25 as compared to the larger co-integrate form in Ab242 as judged by Southern blot analysis (see above) suggest that the recombination reaction is mainly biased toward resolution in the cell (Figure 3).

### Homology between Ab242 plasmids with other *Acinetobacter* plasmids deposited in databases

A BlastN search against the nucleotide GenBank database using each of the three Ab242 plasmid sequences as individual queries indicated no significantly extended homology to deposited plasmid sequences. However, short segments of Ab242 plasmids displaying nucleotide identity values ranging from 84 to 99% with particular regions of other *Acinetobacter* plasmids were detected (Figure S2, Table S6). Notable, many of these homologous regions were found bracketed by XerC/D-like recognition sites (Figure S2).

### Comparative analysis of *bla*_OXA-58_-containing adaptive modules found in *Acinetobacter* plasmids

**Figure 4:**
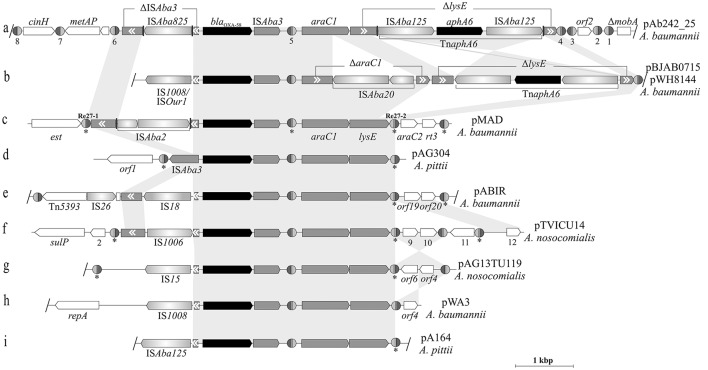
Comparison between *bla*_OXA-58_-containing genetic structures carried by *Acinetobacter* plasmids. Nine *bla*_OXA-58_ containing-adaptive modules carried by *Acinetobacter* plasmids (indicated at the right) and the corresponding genetic contexts in which they are inserted **(a–i)** are depicted, with that found in pAb242_25 (this work) shown at the top. The gray-shaded background interconnecting the different structures highlights the homologous regions (nucleotide sequences ≥ 95% identity). Inverted regions between a given pair of structures are depicted with gray cross sectors. The different CDS and corresponding orientations are denoted, with truncated or interrupted genes indicated by gray boxes with white arrowheads inside indicating their original orientations. The *bla*_OXA-58_ CHDL and *aphA6* aminoglycosides resistance genes are labeled in black. The different IS interrupting the IS*Aba3* element located upstream of *bla*_OXA-58_ are indicated by the designations adopted in the corresponding references. In **(a,b)**, the regions encompassing the composite transposon Tn*aphA6*, their different directions and insertion sites within the *lysE* gene, and the remnants of this gene (Δ*lysE*) in each case are indicated. The inferred XerC/D-like sites are shown as in Figure 2. In **(b–i)**, the sites noted previously by other authors (see references below) are indicated by asterisks below the structures. In the case of pMAD **(c)** the two original XerC/D sites located at the borders of the module are indicated by their original designations Re27-1 and Re27-2, respectively. The slash (/) symbols at the borders of a given structure indicate that further sequence data was provided in databases beyond these positions; otherwise the structure was interrupted at the point where no further sequence information was available. Further details on the XerC/D sites, plasmids, and the *Acinetobacter* species from which they were isolated or characterized are provided in Tables S3, S7 and in references (Poirel and Nordmann, 2006; Zarrilli et al., 2008; D'Andrea et al., 2009; Merino et al., 2010; Grosso et al., 2012; Fu et al., 2014; Blackwell and Hall, 2017).

Figure 4 shows a comparative analysis of the structures and genetic contexts of *bla*_OXA-58_-containing adaptive modules between pAb242_25 and other plasmids of *Acinetobacter* species of the *A. calcoaceticus*/*A. baumannii* complex (see Table S7 for details). These comparisons revealed a common skeleton structure embedded in different genetic contexts in all cases, and which is represented by an IS*Aba3* composite transposon carrying the *bla*_OXA-58_ gene accompanied by *araC1* and *lysE* genes (Poirel and Nordmann, 2006; Evans and Amyes, 2014; Fu et al., 2014; Da Silva and Domingues, 2016). This co-occurrence of *bla*_OXA-58_ with *araC1* has been noted previously, and suggested to play roles in the regulation of the CHDL gene (Fondi et al., 2010). The basic structure described above is reflected in pAG304 from *A. pittii* (Figure 4d) and also in some plasmids from *A. nosocomialis* (Fu et al., 2014). The various IS inserted in the IS*Aba3* copy located upstream of the *bla*_OXA-58_ gene in the modules driving overexpression of the CHDL gene (Table S7) represent most probably recent and independent acquisitions in different hosts selected by carbapenem therapy. Of note, only two of these *bla*_OXA-58_-containing adaptive modules, those carried by pBJAB0715 (Zhu et al., 2013) or pWH8144 (Fu et al., 2014; Figure 4b) and by pAb242_25 (Figure 4a; this work), also carried Tn*aphA6* elements and simultaneously conferred resistance to carbapenems and aminoglycosides to susceptible *Acinetobacter* hosts. However, the different insertion sites and orientations of Tn*aphA6* within *lysE* between them indicate independent acquisitions of this mobile element by the corresponding structures. The evidence above, added to the presence of an additional IS*Aba20* disrupting *araC1* in pBJAB0715 or pWH8144 (absent in pAb242_25), provide additional support of separate histories of IS and transposon acquisitions by each of these modules after their departure from a common ancestral module.

Concerning the XerC/D-like sites associated to these structures, it is noteworthy the existence of three XerC/D-like sites located in alternate inverse orientations in association to the basic module located in pAG304, with two directly-oriented sites bracketing this module and the third located within it (Figure 4d). Two of these sites, the internal one and the site located immediately downstream of *lysE* gene (*i. e*., the equivalents to #5 and #4, respectively, in Figure 4a) are absolutely conserved both in location and orientation among all of these structures (Figure 4). In turn, the XerC/D site located at the left boundary of the module (i.e., the equivalent to #6 in in Figure 4a) is less conserved but still present in many of these structures. The conspicuous absence of the latter site in some structures (e.g., Figures 4b,h,i,) probably resulted from disruptive IS insertions at this region. Of note, the content of XerC/D-like sites outside the limits posed by the genetic structure common to all plasmids also varies substantially between them, with the adaptive module located in pAb242_25 (Figure 4a) representing the most promiscuous case both in number and orientations of XerC/D-like sites.

## Discussion

In this work we characterized in detail the plasmids present in *A. baumannii* Ab242, a MDR clinical strain of the CC104 isolated in Argentina, to obtain clues into different aspects related to plasmid diversity, evolutionary dynamics, and underlying mechanisms of dissemination among the *Acinetobacter* population of genetic structures conferring *bla*_OXA-58_-mediated carbapenem resistance. Sequence analysis indicated the existence of three novel plasmids in Ab242 carrying distinct replication and stability modules and lacking self-transferability functions (Figure 2, Table 1). Only one of them, a bi-replicon plasmid of around 25 kbp in size designated pAb242_25, was found to harbor an adaptive module carrying a *bla*_OXA-58_ gene with an upstream IS*Aba825* insertion which we previously reported to promote the over-expression of the CHDL gene (Ravasi et al., 2011) accompanied by a Tn*aphA6* transposon inserted in a *lysE* gene located downstream of the CHDL gene (Figure 2). Transformation analysis with plasmids isolated from Ab242 indicated that the adaptive module present in pAb242_25 can simultaneously provide resistance to carbapenems and aminoglycosides to an *Acinetobacter* host (Table S1).

Amikacin was amply used in the past to treat infections due to susceptible *A. baumannii* strains, and it was replaced by carbapenem therapy around 20 years ago for strains which have acquired MDR (Limansky et al., 2004; Peleg et al., 2008; Nigro et al., 2011). The *aphA6* gene conferring resistance to amikacin and other aminoglycosides was first described among clinical *Acinetobacter* spp. isolates recovered after 1984 (Lambert et al., 1990). Evidence for transposability was also obtained (Lambert et al., 1990), and it was confirmed later that this gene was in fact part of a composite transposon designated Tn*aphA6* (Nigro et al., 2011). It is not totally unexpected then the finding of *A. baumannii* plasmids carrying Tn*aphA6* and *bla*_OXA_ gene(s) with the ability to confer simultaneous resistance to amikacin and carbapenems, as is the case described here for Ab242 isolated in 1997 (Limansky et al., 2004; Mussi et al., 2011). *Acinetobacter* plasmids with these characteristics can be broadly divided in two groups. On the one hand, self-transferable plasmids such as pD46-3 and pABUH1 with very similar backbones associated to GR6 replicases, and which have acquired separately Tn*aphA6* and *bla*_OXA-23_-containing complex transposons such as Tn*2006* or Tn*2008* (Nigro et al., 2015). On the other hand, plasmids lacking self-transferability such as pAb242_25 (this work), pBJAB07104 (Zhu et al., 2013) or pWH8144 (Fu et al., 2014), which show dissimilar backbones associated to different GR replicases and share *bla*_OXA-58_-containing adaptive modules exhibiting Tn*aphA6* and distinct IS driving *bla*_OXA-58_ overexpression collected on different occasions (Figure 4). The diverse genetic contexts in which these adaptive modules are embedded in the different plasmids, and the lack of self-transferability of the plasmids that carry them pose questions on how these resistance structures were mobilized to these locations and on their dissemination mechanisms among the *Acinetobacter* population (Bertini et al., 2010; Towner et al., 2011; Evans and Amyes, 2014; Da Silva and Domingues, 2016). The absence of mobilization and transfer functions, however, seems not to pose particular barriers to the horizontal gene transfer (HGT) of *Acinetobacter* plasmids (Bertini et al., 2010; Fondi et al., 2010; Towner et al., 2011), suggesting that transformation, transduction, or even non-canonical HGT, represent effective mechanisms for dissemination (Fondi et al., 2010; Da Silva and Domingues, 2016). Less is known on how the above *bla*_OXA-58_-containing modules could be mobilized to other genome locations, and in this context the observations by several authors that these resistance structures are bordered by XerC/D-like sites has led to proposals that this process could be mediated by site-specific recombination (Poirel and Nordmann, 2006; Zarrilli et al., 2008; D'Andrea et al., 2009; Merino et al., 2010; Poirel et al., 2010; Towner et al., 2011; Grosso et al., 2012; Evans and Amyes, 2014; Fu et al., 2014; Da Silva and Domingues, 2016; Blackwell and Hall, 2017). However, whether the XerC/D-like sites located in *A. baumannii* plasmids could be proficient for site-specific recombination, and how they could mediate the mobilization of the associated resistance structures remained obscure.

We showed in this work that at least some XerC/D-like sites present in Ab242 plasmids can constitute active pairs proficient for site-specific recombination, thus providing clues on how the associated *bla*_OXA-58_-containing adaptive modules could be mobilized. We inferred the presence of several XerC/D-like sites not only in pAb242_25 carrying the *bla*_OXA-58_- and Tn*aphA6*-adaptive module described above, but also in the other Ab242 plasmids lacking antimicrobial resistance genes (Figure 2). We also demonstrated that two of these XerC/D-like sites, #7 located in the proximity of the adaptive module in pAb242_25 and #9 in pAb242_12, conform a proficient pair for site-specific recombination mediating the fusion of these two plasmids (Figure 3). Moreover, the two XerC/D sites generated by this fusion also represented a recombinationally proficient sister pair, mediating in this case the intra-molecular resolution of the co-integrate with the regeneration of the original plasmids.

The finding that *Acinetobacter* plasmids contain XerC/D-like sites capable of conforming recombinationally active pairs mediating both fusions and resolutions has a significant impact on the dynamics of these mobile elements and the possibilities of dissemination of resistance structures they carry, and even on the integration of resistance determinants into chromosomal *dif* sites. In principle, it certainly opens the possibility of co-integrate formation between temporarily-coexisting plasmids in which one of the constituents is endowed with self-transferability capabilities, thus allowing the dissemination of “cargo” plasmids to other cells by conduction (Garcillán-Barcia et al., 2011). The rapid resolution of the co-integrates once in the host cell (Figure 3) certainly adds support to this possibility. Also, the generation of co-integrates between different plasmids increases the possibilities of further intra-molecular rearrangements such as resolutions, deletions, and/or inversions (Colloms, 2013) depending on the locations and orientations of the new available pairs of XerC/D recombining sites. The reported influences of sequences of the sites and their immediate DNA contexts on the directionality of the recombining reaction or even on its feasibility (Cornet et al., 1994) certainly adds further levels of complexity to the process.

*Acinetobacter baumannii* strains carrying more than one replicon have been previously noted (Bertini et al., 2010; Towner et al., 2011) suggesting that replicon fusion may be relatively frequent in this bacterial species and even provide some selective advantages for plasmid dissemination. Among them, we already noted above that co-integrate formation may allow conduction of plasmids lacking mobility or transferability functions. Replicon fusion may also expand the host range of the co-integrate by providing establishment and/or stability functions that facilitate a successful establishment into a new host (Sýkora, 1992; Garcillán-Barcia et al., 2011), a situation independent of the mechanism (conjugation, transformation, transduction, non-canonical HGT) employed for co-integrate dissemination. In this context, we observed above that the co-integrate between pAb242_25 and pAb242_12, but not pAb242_25 alone, was the form that could successfully establish into *A. nosocomialis* when this organism was used as a host for transformation. Last, but not less important, similarly to the case of plasmid multimers the formation of co-integrates may favor the rapid accumulation of plasmid variants carrying adaptive mutations particularly under conditions of selective stress (Mazin et al., 1996).

A close look at the dissimilar genetic contexts in which *bla*_OXA-58_-containing adaptive modules are embedded in different *Acinetobacter* plasmids (Figure 4) provides evidence that some of these regions may have derived from XerC/D-mediated recombinational rearrangements. For instance, the gene cluster *orf2*_*sulP* preceded by a XerC/D-like site and located at the left side of the *bla*_OXA-58_-containing module in pTVICU14 (Figure 4f) is very similar in sequence and arrangement to that found in pAb242_12 (Figure S2, this work). This suggests that in pTVICU14 this particular region may have originated from the fusion of two plasmids mediated by a XerC/D sister pair similarly to the case described here. Also, the opposite orientation of the homologous DNA fragments located immediately downstream of *lysE* and bearing *orf6*_*orf4* genes between pTVICU14 (Figure 4f) and pAG13TU119 (Figure 4g) provides evidence for an intra-molecular inversion event mediated by the pair of oppositely-oriented XerC/D-like sites bracketing this region.

It should be kept on mind that, although the possibilities of plasmid shuffling mediated by site specific recombination at XerC/D sites may be large (and even immensurable as the number of potential sites is increased), the whole process is filtered by selection and only a few plasmid structures will eventually take over a large proportion of the population (Garcillán-Barcia et al., 2011). This selection process may range from point mutations that regulate (or impede) the recombinational activity of a given XerC/D site to complete deletions of the site. Again, a detailed comparative look at the genetic context of the *bla*_OXA-58_-containing adaptive modules of Figure 4 provides evidences of this process, as judged both by the differences in XerC/D-like sites content between the different plasmids and the notorious losses of some conserved sites in a number of them.

Further work is in progress to characterize in detail the different XerC/D-like sites located in *A. baumannii* plasmids and to understand their roles in the evolution and dissemination of antimicrobial resistance platforms among the *Acinetobacter* clinical population.

## Author contributions

AV, MC, AL, and JM-B conceived and designed the work. MC performed the experimental work. MC and GR conducted the bioinformatic analysis. AV, MC, AL, JM-B, and GR analyzed the data. AV wrote the manuscript. All authors read and approved the final manuscript.

### Conflict of interest statement

The authors declare that the research was conducted in the absence of any commercial or financial relationships that could be construed as a potential conflict of interest.
