# Supplementary material for: Site-Specific Recombination at XerC/D Sites Mediates the Formation and Resolution of Plasmid Co-integrates Carrying a blaOXA-58- and TnaphA6-Resistance Module in Acinetobacter baumannii
Source: Front Microbiol. 2018 Jan 26;9:66. doi: 10.3389/fmicb.2018.00066 (PMC5790767; doi:10.3389/fmicb.2018.00066)
Supplement: Supplementary file 1 [file Table1.DOCX]

Supplementary Material

**Site-specific recombination at XerC/D sites mediates the formation and resolution of plasmid co-integrates carrying a *bla*_OXA-58_- and Tn*aphA6*-resistance module in *Acinetobacter baumannii***

**María M. Cameranesi, Jorgelina Morán-Barrio, Adriana S. Limansky, Guillermo D. Repizo, and Alejandro M. Viale^*^**

Instituto de Biología Molecular y Celular de Rosario (IBR), Departamento de Microbiología, Facultad de Ciencias Bioquímicas y Farmacéuticas, CONICET, Universidad Nacional de Rosario (UNR), 2000 Rosario, Argentina.

*** Correspondence:** Alejandro M. Viale: viale@ibr-conicet.gov.ar

Table S1. Antimicrobial susceptibility profiles of *A. baumannii* Ab242 and *A. nosocomialis* M2 transformed with Ab242 plasmids as determined with VITEK-2.

|  | **Minimal inhibitory concentration (MIC)** | | |
| --- | --- | --- | --- |
| **Antimicrobial** | Ab242 | *A. nosocomialis* M2 | *A. nosocomialis* M2 transformed with Ab242 plasmids*^a^* |
| Ampicillin | ≥32 | 16 | ≥32 |
| Ampicillin/Sulbactam | 16 | ≤2 | 4 |
| Piperacillin/Tazobactam | ≥128 | ≤4 | ≥128 |
| Cefotaxime | ≥64 | 8 | 8 |
| Ceftazidime | 16 | 4 | 4 |
| Cefepime | 16 | 2 | 2 |
| Imipenem | ≥16 | ≤0.25 | 8 |
| Meropenem | ≥16 | ≤0.25 | 4 |
| Amikacin | ≥64 | ≤2 | ≥64 |
| Gentamicin | ≥16 | ≤1 | ≥16 |
| Kanamycin*^b^* | ≥64 | 2 | ≥64 |
| Ciprofloxacin | ≥4 | ≤0.25 | ≤0.25 |
| Nitrofurantoin | ≥512 | ≥512 | ≥512 |
| Colistin*^c^* | ≤0.25 | ≤0.25 | ≤0.25 |
| Trimethoprim/Sulfamethoxazole (1.25/23.75) | ≥320 | ≤20 | ≤20 |
| Presence of the IS*Aba825*-*bla*_OXA-58_ arrangement*^d^* | + | - | + |

*^a^A. nosocomialis* M2 cells transformed with Ab242 plasmids were selected on LB agar supplemented with 2 µg/ml IPM, and the results of a representative transformant clone are shown.

*^b^*Determined by the macrodilution method using MH broth in accordance to CLSI recommended procedures.

*^c^*Determined by the microdilution method using MH broth in accordance to CLSI recommended procedures.

*^d^*Detected by a PCR assay with specific primers. (+): Detected; (-): Not detected. For details see Materials and Methods.
