# Supplementary material for: Site-Specific Recombination at XerC/D Sites Mediates the Formation and Resolution of Plasmid Co-integrates Carrying a blaOXA-58- and TnaphA6-Resistance Module in Acinetobacter baumannii
Source: Front Microbiol. 2018 Jan 26;9:66. doi: 10.3389/fmicb.2018.00066 (PMC5790767; doi:10.3389/fmicb.2018.00066)
Supplement: Supplementary file 2 [file Table2.DOCX]

Supplementary Material

**Site-specific recombination at XerC/D sites mediates the formation and resolution of plasmid co-integrates carrying a *bla*_OXA-58_- and Tn*aphA6*-resistance module in *Acinetobacter baumannii***

**María M. Cameranesi, Jorgelina Morán-Barrio, Adriana S. Limansky, Guillermo D. Repizo, and Alejandro M. Viale^*^**

Instituto de Biología Molecular y Celular de Rosario (IBR), Departamento de Microbiología, Facultad de Ciencias Bioquímicas y Farmacéuticas, CONICET, Universidad Nacional de Rosario (UNR), 2000 Rosario, Argentina.

*** Correspondence:** Alejandro M. Viale: [viale@ibr-conicet.gov.ar](mailto:viale@ibr-conicet.gov.ar)

Table S2. Oligonucleotide primers used for PCR analysis and primer walking.

| **Primer pairs*^a^*** | | **Hybridization region in plasmid** | **Sequence (5´→3´)** | **Expected product size**  **(bp)** | **Reference or source** |
| --- | --- | --- | --- | --- | --- |
| A | 52-3´F | 22,594-22613 | GGGTTACGTTGCTGATCACA | 1,743 | This work |
|  | 80-5´R | 24,314-24,336 | TGATCGCTATGTCACATACCATG |  | This work |
| B | 52-5´R | 5,760-5,781 | TCAAAGTCATCTAGGTCGATGG | 4,233 | This work |
|  | 80-3´F | 1,549-1,567 | GTCATCAATGCACAGCGGT |  | This work |
| C | OXA-58R | 7,905-7,924 | TACGACGTGCCAATTCTTGA | 957 | Ravasi *et al.* (2011) |
|  | PISAba825F | 8,849-8,865 | ATCCTGACCATAATGTG |  |  |
| D | 58-5´R | 1,687-1,708 | TACTTCGTGTCTATGGTGAGCC | 1,600 | This work |
|  | *metAP*-5´F | 10,012-10,041 | CGCTCAATAAATGCTCAGGCTGCTTTCTGG |  | This work |
| E | *metAP*-5´F | 10,012-10,041 | CGCTCAATAAATGCTCAGGCTGCTTTCTGG | 1,227 | This work |
|  | *cinH-3´* R | 11,210-11,239 | GACCAAGCTGAAGCTTTACGACAAGCATGG |  | This work |
| F | 89-R | 7,560-7,587 | GTATCCCTGTTCCAGAACACGTTGAGCC | 717 | This work |
|  | 67-Fw | 6,870-6,897 | TTCTGGAGCAGCAGCTTGAAATCAACCG |  | This work |
| G | OXA-58-Fw-5´Biot | 8,199-8,225 | TGAATAACTCAATCATCGATCAGAATG | 689 | This work |
|  | OXA-58-Rv-5´Biot | 7,537-7,563 | TTTACGTAGAGCAATATCATCACCAGC |  | This work |

*^a^*See scheme below in Supplementary Figure 2 for the corresponding plasmid regions covered by the PCR amplicons generated by the indicated primer pairs. Some of these primers were individually employed also for primer walking reactions as also indicated in the Figure 2.
