# Supplementary material for: Site-Specific Recombination at XerC/D Sites Mediates the Formation and Resolution of Plasmid Co-integrates Carrying a blaOXA-58- and TnaphA6-Resistance Module in Acinetobacter baumannii
Source: Front Microbiol. 2018 Jan 26;9:66. doi: 10.3389/fmicb.2018.00066 (PMC5790767; doi:10.3389/fmicb.2018.00066)
Supplement: Supplementary file 4 [file Table4.DOCX]

Supplementary Material

**Site-specific recombination at XerC/D sites mediates the formation and resolution of plasmid co-integrates carrying a *bla*_OXA-58_- and Tn*aphA6*-resistance module in *Acinetobacter baumannii***

**María M. Cameranesi, Jorgelina Morán-Barrio, Adriana S. Limansky, Guillermo D. Repizo, and Alejandro M. Viale^*^**

Instituto de Biología Molecular y Celular de Rosario (IBR), Departamento de Microbiología, Facultad de Ciencias Bioquímicas y Farmacéuticas, CONICET, Universidad Nacional de Rosario (UNR), 2000 Rosario, Argentina.

*** Correspondence:** Alejandro M. Viale: viale@ibr-conicet.gov.ar

Table S4. Transposable elements detected in pAb242_25.

| **Mobile element** | **Location (bp)** | **Target site duplication** | **Presence of inverted repeats*^a^*** | |
| --- | --- | --- | --- | --- |
|  |  |  | **IRL** | **IRR** |
| IS*Aba125^b^* | 2,150-3,236 | tac | + | + |
| IS*Aba125^b^* | 4,135-5,221 |  | + | + |
| IS*Aba3 ^c^* | 6,662-7455 | - | + | + |
| ΔIS*Aba3^c^* | 8,349-8,468  9,451-9,757 | - | + | - |
| IS*Aba825* | 8,470-9,444 | aaaaatg | + | + |
| IS*26* | 12,088-12907 | cttaatat | + | + |

*^a^*Inverted repeat left (IRL) and/or inverted repeat right (IRR): (+), present; (-), non-detected

*^b^*These two IS are part of composite transposon Tn*aphA6*.

*^c^*These two IS are part of a composite transposon embedding *bla*_OXA-58_. The IS*Aba3* located upstream of this CHDL gene is disrupted by IS*Aba825* (ΔIS*Aba3*) generating a strong promoter driving *bla*_OXA-58_ overexpression (Ravasi *et al*., 2011).
