# Supplementary material for: Site-Specific Recombination at XerC/D Sites Mediates the Formation and Resolution of Plasmid Co-integrates Carrying a blaOXA-58- and TnaphA6-Resistance Module in Acinetobacter baumannii
Source: Front Microbiol. 2018 Jan 26;9:66. doi: 10.3389/fmicb.2018.00066 (PMC5790767; doi:10.3389/fmicb.2018.00066)
Supplement: Supplementary file 5 [file Table5.DOCX]

Supplementary Material

**Site-specific recombination at XerC/D sites mediates the formation and resolution of plasmid co-integrates carrying a *bla*_OXA-58_- and Tn*aphA6*-resistance module in *Acinetobacter baumannii***

**María M. Cameranesi, Jorgelina Morán-Barrio, Adriana S. Limansky, Guillermo D. Repizo, and Alejandro M. Viale^*^**

Instituto de Biología Molecular y Celular de Rosario (IBR), Departamento de Microbiología, Facultad de Ciencias Bioquímicas y Farmacéuticas, CONICET, Universidad Nacional de Rosario (UNR), 2000 Rosario, Argentina.

*** Correspondence:** Alejandro M. Viale: viale@ibr-conicet.gov.ar

Table S5. Definition of a consensus *Acinetobacter* XerC/D recognition motif for the detection of the equivalent sites in Ab242 plasmids.

| **Site designation** | **Site sequence (5´→3´)** | **Reference** | **GenBank accession numbers** |
| --- | --- | --- | --- |
| *A. baylyi* chromosome *dif* | GATTCGTATAATGTATATTATGTTAAAT | Carnoy *et al*. (2009) | NC_012813.1 |
| Re27_1 (pMAD)  Re27_2 (pMAD) | -TTTCGTATAACCGCCATTATGTTAAAT *^a^*  -TTTCGTATAACAGCCATTATGTTAAAT *^a^* | Poirel *et al*. (2006) | AY665723.1 |
| XerC/D_1 (pAb120)  XerC/D_2 (pAb120)  XerC/D_3 (pAb120)  XerC/D_4 (pAb120)  XerC/D_5 (pAb120) | ATTTCGTATAAGGTGTATTATGTTAATT  ATTTAACATAATGGGCGTTATGCGAAAT  GATTCGCATAAGAGATTTTATGTTAAAT  ACTTCGTATAATCGCCATTATGTTAAAT  GCTTCGCATAAGGTGTATTATGTTAATT | Povilonis  *et al*. (2013) | JX069966.1 |
| XerC/D_1 (pXBB-9)  XerC/D_2 (pXBB-9)  XerC/D_3 (pXBB-9)  XerC/D_4 (pXBB-9) | ATTTCGTATAACACCA-TTATGTTAAAT *^a^*  ATTTCGTGTAATAGAATTTATGTTAAAT  ACTTCACATAAGAAATTTTATGTTAA-- *^a^*  ACTTCACATAAGAAATTTTATGTTAA-- *^a^* | Feng *et al*. (2015) | NZ_CP010351.1 |
| XerC/D_1 (pABVA01)  XerC/D_2 (pABVA01) | ATTTCGTATAACGTGTATTATGTTAATT  ACTTCGTATAATCGCCATTATGTTAAAT | D´Andrea  *et al*. (2009) | NC_012813.1 |
| XerC/D_1 (pMMA2)  XerC/D_2 (pMMA2)  XerC/D_3 (pMMA2) | ACTTCGGATAACGCCCATTATGTTAAAT  -TTTCGTATAAGGTGTATTATGTTAA-- *^a^*  GCTTCGCATAAGAGATTTTATGTTAAAT | Merino *et al*.  (2010) | GQ377752.1 |
| Consensus*^b^* | **atTTcgtaTAAggtgtaTTATGttAAat** |  |  |

*^a^*The missing nucleotides in these cases are represented by hyphens (-) in the alignments.

*^b^*In the consensus sequence the uppercase letters denote a completely conserved nucleotide at a given position in the 17 sequences analyzed.
