# Supplementary material for: Site-Specific Recombination at XerC/D Sites Mediates the Formation and Resolution of Plasmid Co-integrates Carrying a blaOXA-58- and TnaphA6-Resistance Module in Acinetobacter baumannii
Source: Front Microbiol. 2018 Jan 26;9:66. doi: 10.3389/fmicb.2018.00066 (PMC5790767; doi:10.3389/fmicb.2018.00066)
Supplement: Supplementary file 6 [file Table6.DOCX]

Supplementary Material

**Site-specific recombination at XerC/D sites mediates the formation and resolution of plasmid co-integrates carrying a *bla*_OXA-58_- and Tn*aphA6*-resistance module in *Acinetobacter baumannii***

**María M. Cameranesi, Jorgelina Morán-Barrio, Adriana S. Limansky, Guillermo D. Repizo, and Alejandro M. Viale^*^**

Instituto de Biología Molecular y Celular de Rosario (IBR), Departamento de Microbiología, Facultad de Ciencias Bioquímicas y Farmacéuticas, CONICET, Universidad Nacional de Rosario (UNR), 2000 Rosario, Argentina.

*** Correspondence:** Alejandro M. Viale: viale@ibr-conicet.gov.ar

Table 6. Homology between regions of Ab242 plasmids with other *Acinetobacter* spp. plasmids.

| **Plasmid query** | **Position in plasmid and region length (bp)** | **Matches with plasmid** | **Strain carrying the plasmid matching region** | **Nucleotide identity**  **(%)** | **GenBank accession number** |
| --- | --- | --- | --- | --- | --- |
| pAb242_9 | 2,709-3,858 (1,150)  3,930-4,604 (675)  5,731-6,414 (684)*^a^* | pAba3207a  pABIR  pAB2 | *A. baumannii* 3207  *A. baumannii* transconjug. 1  *A. baumannii* ATCC17978 | 99  84  95 | CP015365.1  NC_010481.1  NC_009084.1 |
| pAb242_12 | 2,264-2,811 (548)  2,798-4,961 (2,165) *^a^*  3,150-4,961 (1,813)  5,366-7,208 (1,843) | pMAC  pOXA58-AP_882  pTVICU14  pM131-5 | *A. baumannii* 19606  *A. pittii* AP_882  *A. nosocomialis* TVICU14  *Acinetobacter* sp M131 | 98  95  94  99 | AY541809.1  CP014479.1  NG_040884.1  NC_025171.1 |
| pAb242_25 | 10,924-11,840 (917) *^a^*  13,385-13,962 (577)  16,345-18,773 (2,429) | pACICU1  pACICU1  pD36-4 | *A. baumannii* ACICU  *A. baumannii* ACICU  *A. baumannii* D36 | 98  90  88 | CP000864.1  CP000864.1  CP012956.1 |

*^a^*Flanked with XerC/D-like recognition sites
