# Supplementary material for: Site-Specific Recombination at XerC/D Sites Mediates the Formation and Resolution of Plasmid Co-integrates Carrying a blaOXA-58- and TnaphA6-Resistance Module in Acinetobacter baumannii
Source: Front Microbiol. 2018 Jan 26;9:66. doi: 10.3389/fmicb.2018.00066 (PMC5790767; doi:10.3389/fmicb.2018.00066)
Supplement: Supplementary file 7 [file Table7.DOCX]

Supplementary Material

**Site-specific recombination at XerC/D sites mediates the formation and resolution of plasmid co-integrates carrying a *bla*_OXA-58_- and Tn*aphA6*-resistance module in *Acinetobacter baumannii***

**María M. Cameranesi, Jorgelina Morán-Barrio, Adriana S. Limansky, Guillermo D. Repizo, and Alejandro M. Viale^*^**

Instituto de Biología Molecular y Celular de Rosario (IBR), Departamento de Microbiología, Facultad de Ciencias Bioquímicas y Farmacéuticas, CONICET, Universidad Nacional de Rosario (UNR), 2000 Rosario, Argentina.

*** Correspondence:** Alejandro M. Viale: viale@ibr-conicet.gov.ar

Table 7. Features of *bla*_OXA-58_-containing adaptability modules described in *Acinetobacter* spp.

| **Strain** | **Plasmid**  **size (kbp)** | **Structure listed in Fig. 3** | **IS promoting *bla*_OXA-58_ overexpression*^a^*** | **GenBank accession number** |
| --- | --- | --- | --- | --- |
| *A. baumannii* 242  *A. baumannii* BJAB0715  *A. baumannii* WH8144  *A. baumannii* MAD  *A. pittii* AG304  *A. baumannii* ABIR  *A. nosocomialis* TVICU14  *A. nosocomialis* AG13TU119  *A. baumannii* WA3  *A. pittii* A164 | 25  52  9*^b^*  30*^b^*  5.7*^b^*  29  8.5*^b^*  8.1*^b^*  7.5*^b^*  6.5*^b^* | a  b  b  c  d  e  f  g  h  i | IS*Aba825*  IS*1008*  IS*Our1*  IS*Aba2*  nil  IS*18*  IS*1006*  IS*15*  IS*1008*  IS*Aba125* | KR055667  CP003848.1  JQ241792.1  AY665723.1  JQ241790.1  NC_010481.1  JQ241791.1  JQ241789.1  JQ241791.1  GU911349.1 |

*^a^*Designations of the different IS disrupting the IS*Aba3* copy located upstream of *bla*_OXA-58_.

*^b^*Partial plasmid sequence.
