# Supplementary material for: Site-Specific Recombination at XerC/D Sites Mediates the Formation and Resolution of Plasmid Co-integrates Carrying a blaOXA-58- and TnaphA6-Resistance Module in Acinetobacter baumannii
Source: Front Microbiol. 2018 Jan 26;9:66. doi: 10.3389/fmicb.2018.00066 (PMC5790767; doi:10.3389/fmicb.2018.00066)
Supplement: Supplementary file 8 [file Image1.PDF]

Supplementary Material

Site-specific recombination at XerC/D sites mediates the formation and resolution of plasmid co-integrates carrying a *bla*$_{OXA-58}$- and Tn*aphA6*-resistance module in *Acinetobacter baumannii*

María M. Cameranesi, Jorgelina Morán-Barrio, Adriana S. Limansky, Guillermo D. Repizo, and Alejandro M. Viale*

Instituto de Biología Molecular y Celular de Rosario (IBR), Departamento de Microbiología, Facultad de Ciencias Bioquímicas y Farmacéuticas, CONICET, Universidad Nacional de Rosario (UNR), 2000 Rosario, Argentina.

*** Correspondence:** Alejandro M. Viale: viale@ibr-conicet.gov.ar

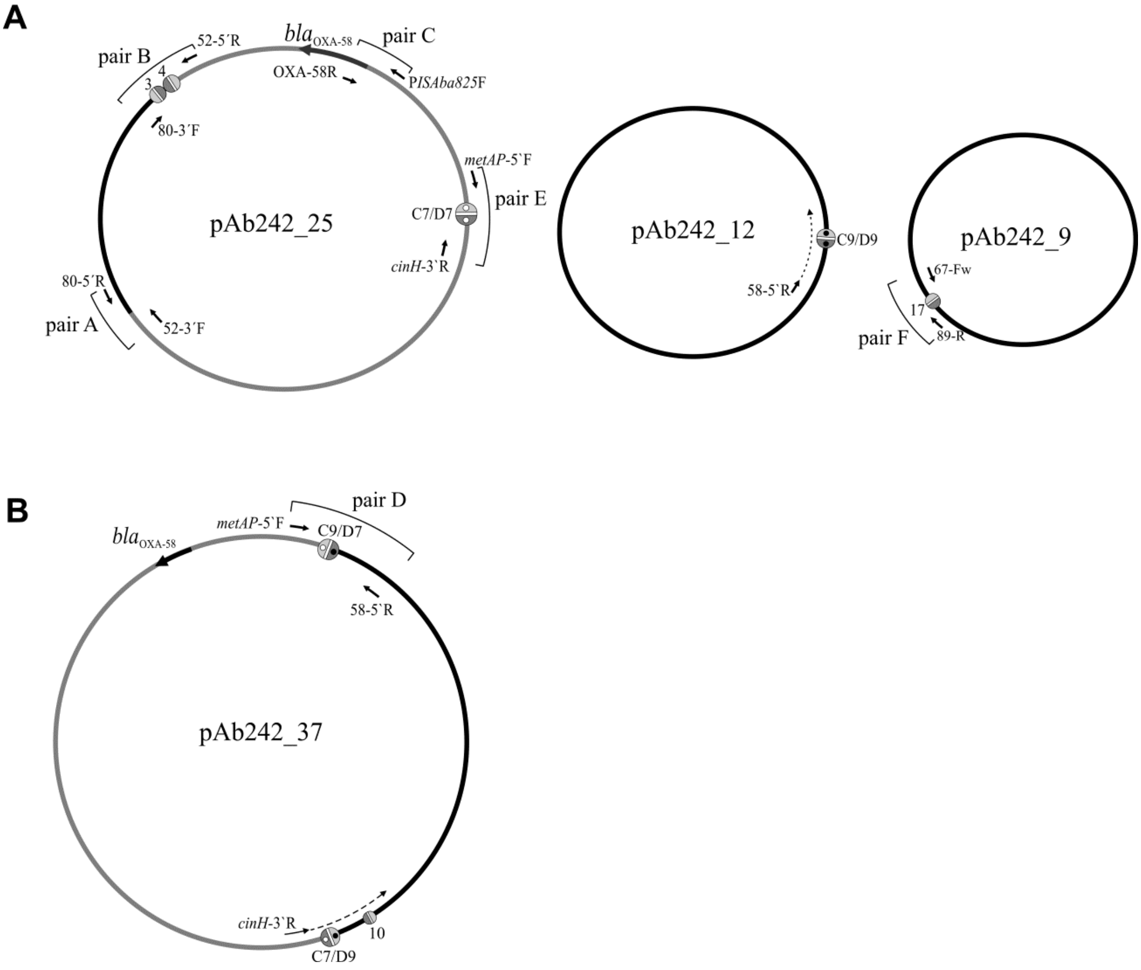

Figure S1. Schematic representation of Ab242 plasmids showing the gap closure strategy used and the confirmation of pAb242_37 cointegrate. (A) Reconstruction of plasmids pAb242_25, pAb242_12, and pAb242_9. The circular forms of each of these plasmids was inferred from the corresponding assembled contigs obtained from the pyrosequencing data followed by PCR with specific primer pairs and primer walking analysis conducted on extracted Ab242 plasmids. The primer details are indicated in Supplementary Table 5. The two contigs obtained by pyrosequencing in the case of pAb242_25 (the corresponding extensions are indicated in black and gray, respectively) were joined using PCR pairs A and B followed by sequencing of the corresponding amplicons. The locations and orientations of *metAP* and *cinH* and the presence of XerC/D recognition site #7 in the intergenic region separating these genes were verified also using PCR pair E. The plasmid region covered by the amplicon generated by PCR pair C in pAb242_25 used to verify the presence of the IS*Aba825*-*bla*$_{\mathrm{OXA\text{-}58}}$ arrangement is also indicated. In the case of pAb242_12 and pAb242_9, the extremes of the single contigs obtained by pyrosequencing in each case were joined by primer walking and PCR using pair F, respectively. The different XerC/D sites identified in the above analyses are highlighthed as ovals (not drawn to scale), with the XerC and XerD recognition sequences depicted as dark gray and light gray semi-ovals, respectively. The XerC_7/XerD_7 and XerC_9/XerD_9 sites active in recombination (this work) have been arbitrarily enlarged, with the recognition sites for the XerC and XerD recombinases additionally decorated with open and closed inner circles within, respectively, to facilitate the visualization of the fusions generating XerC_9/XerD_7 (C9/D7) and XerC_7/XerD_9 (C7/D9) in pAb242_37 (see below). **(B)** Strategy used to confirm the fusion between pAb242_25 and pAb242_12 at the sister pair XerC_7/XerD_7 and XerC/D_9. The sectors corresponding to pAb242_25 and pAb242_12 in this co-integrate are highlighted in gray and black, respectively. The XerC_9/XerD_7 fusion was verified by PCR using primer pair D followed by amplicon sequencing, while the XerC_7/XerD_9 fusion was confirmed by primer walking. For additional details see Table 2 and Figure 3.
