# Supplementary material for: Site-Specific Recombination at XerC/D Sites Mediates the Formation and Resolution of Plasmid Co-integrates Carrying a blaOXA-58- and TnaphA6-Resistance Module in Acinetobacter baumannii
Source: Front Microbiol. 2018 Jan 26;9:66. doi: 10.3389/fmicb.2018.00066 (PMC5790767; doi:10.3389/fmicb.2018.00066)
Supplement: Supplementary file 9 [file Image2.PDF]

Supplementary Material

Site-specific recombination at XerC/D sites mediates the formation and resolution of plasmid co-integrates carrying a *bla*$_{OXA-58}$- and Tn*aphA6*-resistance module in *Acinetobacter baumannii*

María M. Cameranesi, Jorgelina Morán-Barrio, Adriana S. Limansky, Guillermo D. Repizo, and Alejandro M. Viale*

Instituto de Biología Molecular y Celular de Rosario (IBR), Departamento de Microbiología, Facultad de Ciencias Bioquímicas y Farmacéuticas, CONICET, Universidad Nacional de Rosario (UNR), 2000 Rosario, Argentina.

*** Correspondence:** Alejandro M. Viale: viale@ibr-conicet.gov.ar

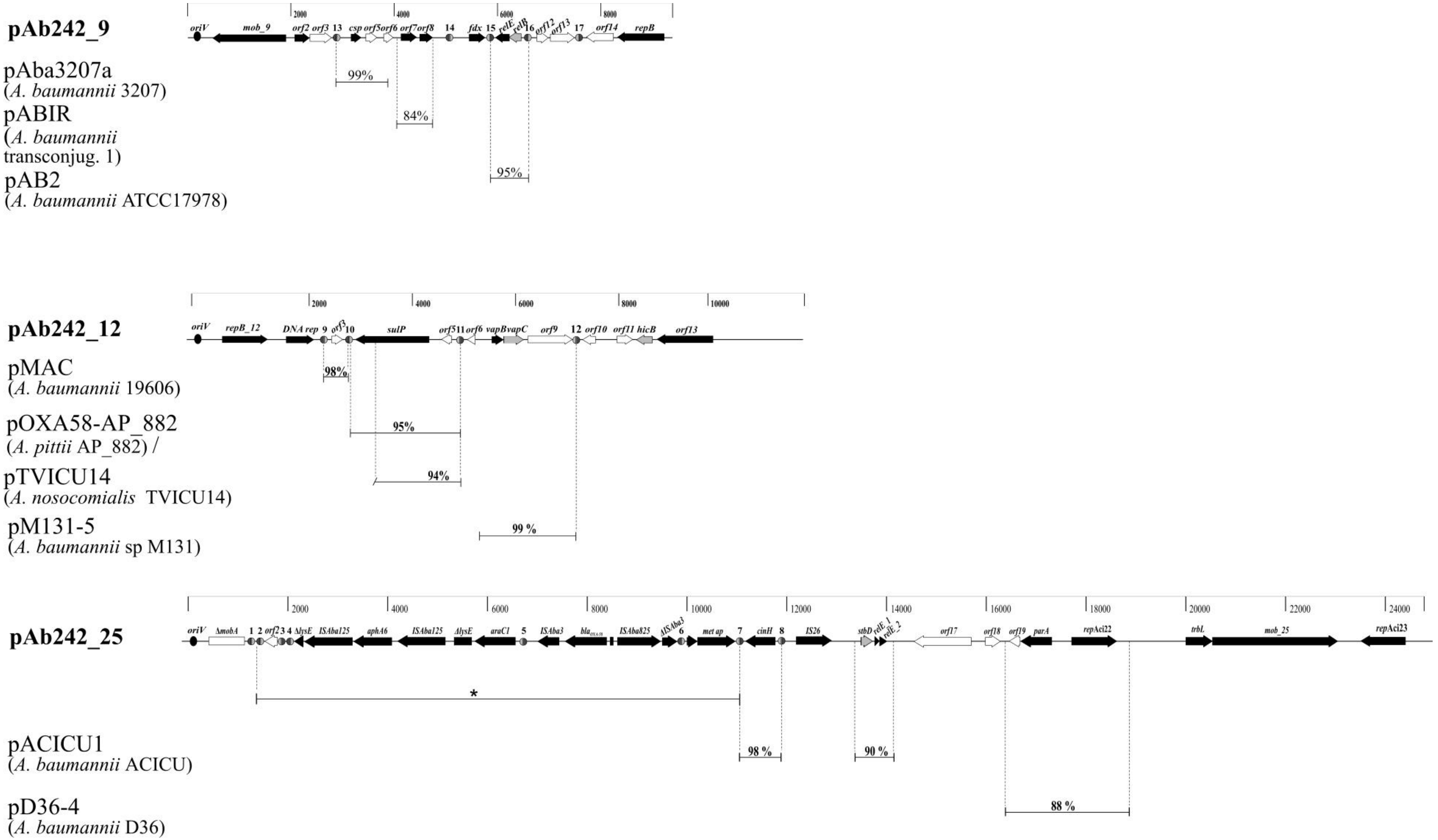

pAb242_9
pAba3207a
(A. baumannii 3207)
pABIR
(A. baumannii
transconjug. 1)
pAB2
(A. baumannii ATCC17978)
2000
4000
6000
8000
oriV
mob_9
orf2
orf3
13
csp
orf5
orf6
orf7
orf8
14
fdx
15
relE
relB
16
orf12
orf13
17
orf14
repB
99%
84%
95%
pAb242_12
pMAC
(A. baumannii 19606)
pOXA58-AP_882
(A. pittii AP_882) /
pTVICU14
(A. nosocomialis TVICU14)
pM131-5
(A. baumannii sp M131)
2000
4000
6000
8000
10000
oriV
repB_12
DNA rep
9
orf3
10
sulP
orf5
11
orf6
vapB
vapC
orf9
12
orf10
orf11
hicB
orf13
98%
95%
94%
99 %
pAb242_25
pACICU1
(A. baumannii ACICU)
pD36-4
(A. baumannii D36)
2000
4000
6000
8000
10000
12000
14000
16000
18000
20000
22000
24000
oriV
ΔmobA
1
2
orf2
3
4
ΔlysE
ISAba125
aphA6
ISAba125
ΔlysE
araC1
5
ISAba3
ISAba825
ΔISAba3
6
met ap
7
cinH
8
IS26
stbD
relE_1
relE_2
orf17
orf18
orf19
parA
repAci22
trbL
mob_25
repAci23
*
98 %
90 %
88 %

Figure S2. Lineal map of Ab242 plasmids showing homology regions with other *Acinetobacter* plasmids. The regions of homology and the percentage of nucleotide identity between the Ab242 plasmids sequences with those of other *Acinetobacter* plasmids are shown. The identified ORFs and the location of the XerC/D-like sites (this work) in each of these plasmids are also indicated. The XerC and XerD binding regions are depicted as dark and gray semi-ovals (not drawn to scale), respectively. The region spanning the adaptability module in pAb242_25 is indicated with an asterisk (*). Sizes (in kbp) starting arbitrarily from the *oriV* are shown above each plasmid. Denominations and sources of plasmids are indicated at the left. For further details see Figure 2, Table 2, and Supplementary Table S3).
